# Ocular Trauma among Patients Visiting the Outpatient Department of Ophthalmology in a Tertiary Care Centre

**DOI:** 10.31729/jnma.8368

**Published:** 2023-12-31

**Authors:** Anjila Basnet, Grish Dhakal, Kiran Ghising, Samyam Bickram Pathak, Trishna Shrestha

**Affiliations:** 1 Department of Ophthalmology, KIST Medical College and Teaching Hospital, Mahalaxmi, Lalitpur, Nepal; 2 KIST Medical College and Teaching Hospital, Mahalaxmi, Lalitpur, Nepal; 3 Department of General Practice and Emergency Medicine, KIST Medical College and Teaching Hospital, Mahalaxmi, Lalitpur, Nepal

**Keywords:** *blunt injuries*, *injury*, *ophthalmology*, *prevalence*

## Abstract

**Introduction:**

Ocular trauma is an important cause of blindness and ocular morbidity worldwide. Knowledge of the cause of eye injuries can guide preventive strategies and optimize management capacity. The aim of the study was to find out the prevalence of ocular trauma among patients visiting the outpatient Department of Ophthalmology in a tertiary care centre.

**Methods:**

A descriptive cross-sectional study was conducted among patients with ocular trauma from 10 January 2023 to 5 July 2023 after obtaining ethical approval from the Institutional Review Committee. A convenience sampling method was used. The point estimate was calculated at a 95% Confidence Interval.

**Results:**

Among 4,959 patients, 107 (2.16%) (1.76-2.56, 95% Confidence Interval) had ocular trauma. Among them, 24 (22.43%) had occular trauma due to fall injuries. Ocular trauma was observed in 82 (76.64%) males and the common agent was blunt objects seen in 56 (52.34%).

**Conclusions:**

The prevalence of ocular trauma among patients was lower than in other studies done in similar settings.

## INTRODUCTION

Ocular trauma is an important cause of blindness and ocular morbidity worldwide. In Nepal, retinal diseases were the third most common leading cause of blindness in the survey done in 1981.^1^

Major causes of ocular trauma in Nepal include agricultural and domestic work, road traffic accidents and physical assault.^2^ In Nepal, with a developing economy, poor health facilities and poor access to the health care system, trauma is a significant cause of ocular morbidity. Knowledge of the causes of eye injuries can aid in guiding preventive strategies and optimizing management capacity. Public awareness and strict legislation to use personal protective devices such as safety glasses at work can help to reduce the occurrence of work-related ocular injuries.

The aim of this study was to find out the prevalence of ocular trauma among patients visiting the outpatient Department of Ophthalmology in a tertiary care centre.

## METHODS

A descriptive cross-sectional study was conducted in the outpatient Department of Ophthalmology at KIST Medical College and Teaching Hospital (KISTMCTH), Mahalaxmi, Lalitpur, Nepal from 10 January 2023 to 5 July 2023. Ethical approval was taken from the Institutional Review Committee (Reference number: 2079/80/74). All the patients giving consent irrespective of age and gender were included in the study. Patients with life-threatening conditions requiring life support and those unwilling or unable to undergo ocular evaluations were excluded from the study. A convenience sampling method was used. The sample size was calculated using the following formula:


$$\begin{array}{ll} \text{n} & ={\text{Z}}^{2}\times \frac{\text{p}\times \text{q}}{{\text{e}}^{\text{2}}}\\ & =1.96^{2}\times \frac{0.094\times 0.906}{0.01^{2}}\\ & =3,272 \end{array}$$

Where,

n = minimum required sample size
Z = 1.96 at 95 % Confidence Interval (CI)
p = prevalence taken from a previous study, 9.40%^3^
q = 1-p
e = margin of error, 1%

The calculated sample size was 3,272. However, 4,959 patients were included in the study.

Data were collected in proforma including demography details, causes of ocular injury, agent of injury, place of trauma, ocular injury to the time of presentation and place of first aid treatment. A complete ophthalmologic evaluation was done including presenting visual acuity, measured with internally illuminated Snellen's chart. Detailed anterior segment evaluation was done with slit lamp biomicroscopy. The posterior segment evaluation was done with direct and indirect ophthalmoscopes (Heine/Welch Allyn/Volk 90D Aspheric lens/Neitz). Relevant investigations like Ultrasound B scan, X-ray orbit/skull, computed tomography (CT) scan and magnetic resonance imaging (MRI) were done whenever indicated. An ophthalmologist examined all the patients and appropriate intervention was taken. Mechanism of ocular injury is categorized as mechanical and non-mechanical. Mechanical injuries are further classified according to 'Birmingham Eye Trauma Terminology (BETT) by Kuhn and associates into closed-globe and open-globe injuries.^4^

Data was entered and analyzed in Microsoft Excel 2016. The point estimate was calculated at a 95% CI.

## RESULTS

Out of 4,959 patients, 107 (2.16%) (1.76-2.56, 95% CI) had ocular trauma. Among them, 24 (22.43%) were fall injuries. A total of 82 (76.64%) were male and 25 (23.36%) female. The majority of eye injuries occurred at home 42 (39.25%) (Table 1).

**Table 1:** Distribution and clinical profile of ocular trauma (n= 107).

Variables		n {%)
**Causes of ocular Trauma**	Fall injury	24 (22.43)
	Welding arc	14(13.08)
	Physical assault	13 (12.15)
	Road traffic accidents	12 (11.21)
	Sports Injury	11 (10.28)
	Chemical	8 (7.48)
	Wood	6 (5.61)
	Foreign body	5 (4.67)
	Finger	5 (4.67)
	Dust	4 (3.74)
	Insect bite	3 (2.80)
	Plant	2 (1.87)
**Place of trauma**	Home	42 (39.25)
	Road	25 (23.36)
	Construction site	13 (12.14)
	Factory	9 (8.41)
	School	8 (7.48)
	Workplace	6 (5.61)
	Field	4 (3.74)

**Figure 1:**
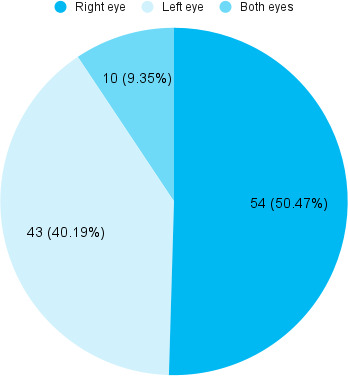
Laterality of affected eye (n= 107).

Out of 107 patients, 54 (50.46%) had affected right eye (Figure 1).

**Figure 2:**
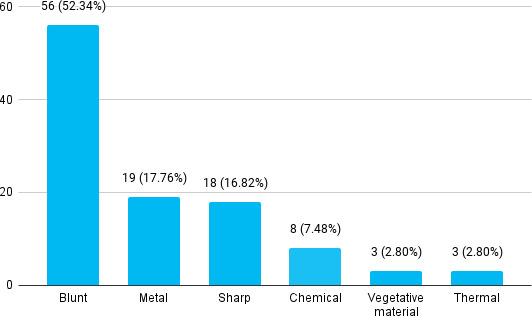
Agent of trauma for ocular injuries (n= 107)

Most injuries were secondary to blunt objects 56 (52.33%) followed by metal 19 (17.75%) (Figure 2).

Most affected age group were young adults between 20-30 years with 27 (25.23%) patients (Table 2).

**Table 2:** Socio-demographic variables of the patients (n= 107).

Variables		n (%)
Age group (years)	0-10	10 (9.35)
	10-20	16 (14.95)
	20-30	27 (25.23)
	30-40	22 (20.56)
	40-50	16 (14.95)
	50-60	6 (5.61)
	>60	10 (9.35)
Ethnicity	Brahman	17 (15.89)
	Chhetri	22 (20.56)
	Newar	21 (19.63)
	Mangolian	23 (21.50)
	Madhesi	15 (14.02)
	Others	9 (8.41)
Occupation	Student	23 (21.50)
	Manual labour	21 (19.63)
	Desk job	21 (19.63)
	Homemaker	16 (14.95)
	Factory job	11 (10.28)
	Retired	8 (7.48)
	Farmer	5 (4.67)
	Unemployed	2 (1.87)
Education level	Primary	27 (25.23)
	Secondary	35 (32.71)
	College Higher	31 (28.97)
	Illiterate	14 (13.08)
Geographic distribution	Hilly	90 (84.11)
	Terai (low land)	17 (15.89)

Among 107 patients, 42 (39.25%) were presented within 6 hours of eye injury and a large proportion of cases had subconjunctival haemorrhage 40 (37.38%) (Table 3).

**Table 3:** Time of presentation and impact of ocular injury among trauma eyes (n= 107).

Variables		n (%)
Duration of ocular injury to the time of presentation (hours)	<6	42 (39.25)
	6-12	8 (7.48)
	12-24	22 (20.56)
	24-48	10 (9.35)
	>48	25 (23.36)
Place of first aid treatment	Hospital	54 (50.47)
	Home	28 (26.17)
	None	19 (17.76)
	Workplace	6(5.61)
Ocular impact of trauma		
Orbit	Orbital wall fracture	3 (2.80)
Eyelid	Eyelid oedema and ecchymosis	25 (23.36)
	Abrasion	13 (12.15)
	Laceration	9 (8.41)
Conjunctiva	Subconjunctival haemorrhage	40 (37.38)
	Hyperemia	31 (28.97)
	Foreign body	5 (4.67)
	Laceration	4 (3.74)
Cornea	Foreign body	23 (21.50)
	Abrasion	15 (14.02)
Uvea	Traumatic Uveitis	6 (5.61)
	Iridodialysis	1 (0.93)
	Conservative	19 (17.76)
Management	Medical	76 (71.03)
	Surgical	11 (10.28)
	Leave against medical advice	1 (0.93)

## DISCUSSION

Among 4,959 patients, 107 (2.16%) had ocular trauma. The prevalence of ocular trauma among patients was lower than in other studies done in similar settings In this study, the most common agent for ocular trauma was blunt object 52.33% and the frequently affected age group were young adults between 20-30 years which was almost similar to studies conducted in Israel.^5^ Ocular injury is a common problem of global concern. Many young adults and children sustain ocular trauma during sports and recreational work in the Western Hemisphere, but in our country, younger adults and children are injured in agricultural work.^6^ Ocular trauma was commonly seen in boys compared with girls and the right eye was affected more frequently than the left eye which was similar to other studies.^7^

In this study the most common cause for ocular trauma was fall injury 22.42% which was higher than the study done in Western Nepal.^8^ A study done in Eastern Nepal concluded that the students were the most vulnerable group for ocular trauma which was similar to this study.^9^ Likewise in a study done in Duwakot, the posterior segment involvement was 31.2% which was higher than the present study.^10^ In a study done in Dhulikhel, a household was the most commonest place of injury which was similar to the present study.^11^ Meanwhile, one study showed that post-traumatic corneal ulceration can be prevented by topical application of 1% chloramphenicol ophthalmic ointment in a timely fashion to the eyes of individuals who have suffered a corneal abrasion in a rural setting.^12^ In the present study, early presentation within 6 hours was found in 39.25% and subconjunctival haemorrhage was the most common ocular finding which was similar to other studies.^13^

The limitation of this study was that the application of occupational eye protections that can help to reduce the occurrence of ocular injury was not assessed. This was a hospital-based cross-sectional study. So, the findings of the present study cannot be extrapolated to the entire population.

## CONCLUSIONS

The prevalence of ocular trauma among patients was lower than in other studies done in similar settings. Public awareness, strict legislation for the use of personal protective eyewear and immediate management of ocular trauma could be made to reduce the occurrence of work-related ocular injuries.
